# Initial Exploration of Canola Producers' Approaches in Response to Changing Climate in the Canadian Prairie Provinces

**DOI:** 10.1002/pei3.70107

**Published:** 2026-01-04

**Authors:** Yohanne Larissa Gavasso‐Rita, Simon Michael Papalexiou, Yanping Li, Amin Elshorbagy, Corinne Schuster‐Wallace

**Affiliations:** ^1^ Department of Civil, Geological, and Environmental Engineering University of Saskatchewan Saskatoon Saskatchewan Canada; ^2^ Global Institute for Water Security University of Saskatchewan Saskatoon Saskatchewan Canada; ^3^ Institute of Global Water Security Hamburg University of Technology Hamburg Germany; ^4^ Institute for Water, Environment and Health United Nations University Hamilton Ontario Canada; ^5^ Department of Physics and Astronomy Western University London Ontario Canada; ^6^ Department of Geography and Planning University of Saskatchewan Saskatoon Saskatchewan Canada

**Keywords:** beneficial management practices, climate change, perceptions

## Abstract

Alberta, Manitoba, and Saskatchewan, the Prairie Provinces of Canada, lead national oilseed cultivation. Canola is a staple crop that provides food oil and feedstock for biofuels. Canola production is vulnerable to climate variability, and climate change has altered crop cycles and affected Canadian canola producers. This study aims to generate an initial understanding of canola producers' perceptions of climate change, their current adaptation strategies, and drivers and barriers to implementing new adaptation strategies in the Canadian Prairies. Besides, identifying the public policy needs to improve the canola production sector. Data were collected through an online survey and key informant interviews. Most participants identified changes in climate and frequency of extreme events. They identified a higher occurrence of heat waves and wind gusts and had to adapt to a higher presence of pests and diseases. Despite climate variability, canola productivity has improved in the last ten years, attributed to better technology and management of inputs. Genetic improvement is seen as a crucial part of canola's resistance to biotic and abiotic events. Most participant producers make independent decisions regarding adaptation and best practices at the field level. There is a vast and diverse outreach from researchers and specialists that producers are able to use in decision‐making around implementing new or improved technologies. Participants recommended new and enhanced public policies to regulate the canola industry and seed market, technologies and data use, fossil fuel use, land and water management, and crop nutrition. These initial understandings point to ways in which regulatory bodies and specialists can continue to support producers to mitigate negative impacts of a changing climate and inform shareholders and policymakers of current needs and expectations.

## 1 Introduction

Canola (Canadian oil, low acid) is one of the most important field crops for agriculture and the economy in Canada (Statistics Canada 2023) and one of the healthiest oils for human consumption (Barthet 2015; Lin et al. 2013). It is cultivated in many continents (FAO 2022), mainly in North America, Europe, and Asia (Raman et al. 2019). Canola production and its associated supply chain has positively impacted the Canadian economy, promoting employment and revenue from seed development, farming, and oil production for consumption and biodiesel for end users (LMC International 2020) as well as export to international markets. Canadian canola production mainly occurs in the Prairie Provinces, Alberta, Manitoba, and Saskatchewan (Agriculture and Agri‐Food Canada 2023a). Canola production totalled 17.8 M metric tons in 22 M seeded acres in 2024. In the same year, the share of production was 16%, 29%, and 55% for Manitoba, Alberta and Saskatchewan, respectively (Statistics Canada 2025). Even though Canada is a large canola producer, climate change and extreme temperatures may directly impact the vegetable oil and biofuel industries (Raman et al. 2019).

Canola production is highly vulnerable to climate variability and extreme events (Raman et al. 2019), which dictate crop production, especially for rainfed systems. Climatic conditions affect canola yield during the growing season on the Canadian Prairies, especially at seed filling stage (Brandt and McGregor 1997). Evidence shows that air temperatures are rising and altering precipitation in the Canadian Prairies (Qian et al. 2019). A lack of water combined with increasing temperatures interferes with food production (Brandt and McGregor 1997; Sauchyn et al. 2020) through higher incidence of pest attacks, disease occurrence, biodiversity losses, and modification of soil fertility and plant physiology (Oishy et al. 2025). Correlations have also been identified between crop yield losses and the escalation of local food insecurity (Karki et al. 2020). Effects of reduced productivity also mean socio‐economic impacts on producers themselves, which in turn can result in decisions that alter interactions between food, energy, and water (Wu et al. 2021).

While numerous research has explored the impacts of a changing climate on crop production, a critical gap remained as the disconnect between scientific research and producer realities. This study brings a novel contribution by bridging this gap, focusing on how Canadian canola producers perceive changes in climate and the underlying factors influencing their adoption or rejection of adaptation measures. As such, the objective of this paper is to share results of an initial exploration of self‐reported changes in climate and water resources, adoption of adaptation strategies, factors that influence decision‐making in responding to a changing climate, and beneficial practices and policies required to support current and future canola production on the Canadian Prairies.

## 2 Methods

Qualitative and quantitative data were collected through a voluntary online survey and key informant interviews. Ethics approval for the study was received through the University of Saskatchewan Behavioral Research Ethics Board (certificate # BEH 4413). Eligible survey participants were over 18 years of age, canola producers for at least one season, and living in Alberta, Saskatchewan, or Manitoba. Survey participants were invited through recruitment poster media in university portals, social media, and emails to canola growers' associations in the Prairie Provinces. The survey collected general information about the farm, experiences with a changing climate and extreme events, climate‐smart farming, facilitators and barriers to adaptation, and demographic and socioeconomic information. The online survey instrument is in the Supporting Information and the responses received are in Table S1. Key informants were 18 or over and were employed in government, professional organizations (e.g., provincial canola grower associations), oilseed and farm management specialists, or researchers. More than 60 recruitment emails were sent to subject‐matter experts with an invitation to participate as a key informant. Interviews lasted between 30 and 60 min and explored changing climate impacts on canola production and adaptation strategies in the Canadian Prairie Provinces, including canola productivity, climate‐smart farms, agricultural adaptation strategies, producer and market preparedness to yield losses, public policies, and future expectations for the canola sector. The questions used in the key informant interviews are in the Supporting Information and the responses received are in Table S2.

### 2.1 Data Analysis

Data were analyzed using basic and descriptive statistical analysis, including correlation and statistical significance (*p* ≤ 0.05). Qualitative responses were analyzed by applying thematic analysis using Nvivo (Woolf and Silver 2018). Thematic analysis (TA) is a method to process qualitative datasets through data coding and theme development (Braun and Clarke 2021). The process follows familiarization with the dataset, data coding, and theme development (initial generation, revision, improvement, definition, and naming) (Braun and Clarke 2021). The code set developed through this process is presented in Table 1.

**TABLE 1:** Final code tree for analysis.

Primary theme (parent node)	Secondary theme (child node)	Tertiary theme (grandchild node)
Adaptation	Climate smart farm	Investment
	Best practices	Climate stress
		Cover crops
		Crop rotation
		Intercropping
		Pest and disease control
		Seeding
		Tillage
		Water management
		Weed control
		Yield potential
	Digital or precision	
	Environmental	
	Genetics	
	Research	
Extreme events	Drought	
	Flood	
	Forecast	
	Frost	
	Hail	
	Heatwave	
	Rainstorm	
Changing climate	Dry	
	Less change	
	More change	
	Rain	
	Snow	
	Temperature	
	Uncertainty	
Impact	Canola	Positive
		Negative
	Economy	Positive
		Negative
	Preparedness	Producer
		Market
Future	Advice	
	Canola	
	Concerns	
	Innovation	
	Need	
*Public policies*		
Outreach	Communication	E‐mail
		Newsletter
		Phone
		Social media
		Website
	Extension events	Field day
		Research presentations
	Reports	
	Response	

## 3 Results and Discussion

A total of 16 canola producers answered the online survey and nine key informants (KI) participated in the interviews. An initial target of 25 participants was established for the KI interviews, but recruitment was stopped at nine as data saturation was achieved (Islam and Aldaihani 2022). While survey responses were much lower than anticipated, responses have been included alongside the KI analyses as collectively they provide a window into the intersection between climate change, canola production, and readiness to adapt.

KIs represented applied researchers, specialists in oilseed, diversification, agronomy, and farm management, research managers, and scientists. All survey and KI participants who identified level of education had a university certificate or diploma, and most held a bachelor's degree. Just under half (46%) of survey participants self‐identified as men and the majority of participants (67%) were over 40 years of age (Table 2). Over half of survey participants described that their farms are part of a corporation (54%), while 31% have a sole proprietorship and 15% have a partnership. Almost 40% of the participant farms were reported as smaller than 2000 acres. Nearly 50% have been farming for over 15 years, and 70% have produced canola for over 9 years (Table 2). All participant farms operate with commercial objectives. Their scope of operation was described as within the municipality (23%), province (77%), Prairies (31%), Canada (23%), or internationally (15%). Almost three quarters (70%) of producers identified oilseeds as their most profitable activity and a top economic performer, while just over three quarters (77%) classified canola as their primary source of farm income.

**TABLE 2:** Summary of survey participants according to farm and economic activity description (*n* = 13).

	Age (years)						Gender identification			Period producing canola (years)			
	18–29	30–39	40–49	50–59	60–69	No response	Man	Woman	No response	1–3	4–6	7–9	> 9
*Farm structure*													
Sole Proprietorship	1			1		2	1		3	1	2		1
Corporation	1	1	1	2	1	1	4	2	1		1		6
Partnership				1			1						1
Unidentified						1			1				1
*Degree of decision‐making over the farm*													
Fully independent	2			4	1	1	5	1	2		2		6
Semi‐dependent		1	1			3	1	1	3	1	1		3
*Farm size (acres)*													
1–2000	1	1		2		1	1	2	2	1	2		2
2001–4000	1			1	1	1	3		1				4
4001–6000			1	1		1	2		1		1		2
6001–8000						1			1				1
*Most profitable farming activity*													
Grains	1					1	1		1		1		1
Oilseeds	1	1	1	2	1	3	4	2	3	1	1		7
Pulses				2			1		1		1		1

### 3.1 Experiences With Extreme Events

**FIGURE 1:**
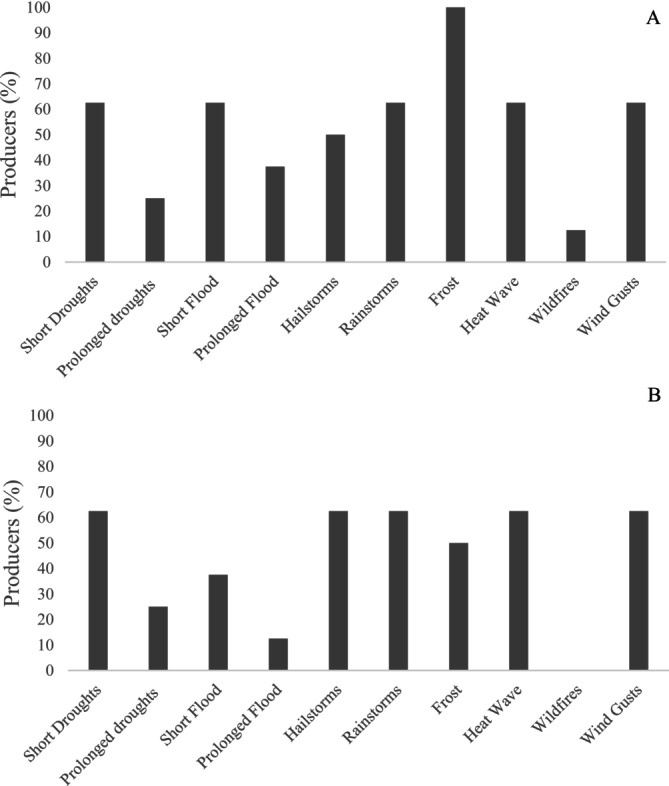
Percentage of participant producers who have experienced extreme events in the last 10 years during the planting season (A) and the harvest season (B).

When asked about experiences with different types of adverse weather events during planting (1A) and harvest (1B), all survey respondents reported having experienced frost in the planting season and half indicated having experienced frosts in the harvest season in the previous 10 years (Figure 1). At least 60% of respondents also reported that they had experienced short droughts and floods, rainstorms, heatwaves, and wind gusts during planting season and short droughts, hail and rainstorms, heatwaves, and wind gusts during planting season (Figure 1).

**FIGURE 2:**
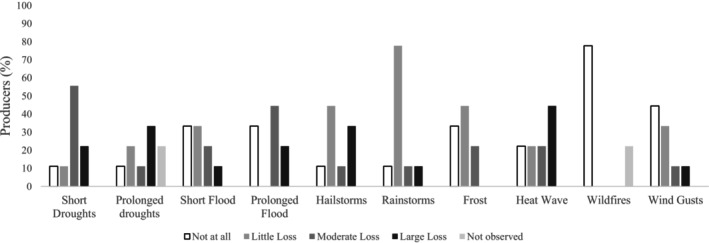
Percentage of participant producers who have experienced an impact on canola production in growth and yield losses from extreme event occurrence.

Spring canola is more susceptible to freezing than winter canola (Wrucke et al. 2020). Still, participants declared little to no impact on canola production from frost (Figure 2). Most producers, however, did report moderate or large losses due to short and prolonged droughts, respectively (Figure 2). According to a KI, “Canola can tolerate a little bit extra moisture, but it can't tolerate droughts for a long time.” [Participant I3]. The climate of the Canadian Prairies is semi‐arid, with highly variable but low annual precipitation and extreme temperatures (McGinn 2010). In 2021, the region suffered from an intense and prolonged drought. It impacted agriculture, grasslands, hydrology, and ecology across the Prairies to varying degrees (Agriculture and Agri‐Food Canada 2021a). A KI noted, “If you don't get any rain for 30 days, you're going to have less than 50% of the production.” *[Participant I3]*. Droughts have cost billions of dollars in crop losses and are the costliest event in the Prairies (Sauchyn et al. 2020). Still, 90% of the participant producers have never used an irrigation system, mainly because of its cost.

Large losses were also associated with heat waves, hailstorms, and floods (Figure 2). Even across the prairies, geographic patterns emerge, with participants reporting more moderate losses in Saskatchewan and larger losses in Manitoba from short droughts (0.92, *p*‐value 0.0004) and moderate to large losses in Saskatchewan from prolonged droughts (0.83, *p*‐value 0.005) that were not observed in Manitoba. Heat waves were associated with large losses in Manitoba (0.67, *p*‐value 0.046), but not in Saskatchewan. This is supported by KI's:

In [Province], we have a bit of a double‐edged sword in the sense that we can have both excess moistures, as well as lack of moisture events. I would also add that, in July, having the very high heat that we've been seeing in the past couple of years is affecting our flowering and causing abortion of the flowers, and that will affect our final yield, as well. Also, one minor, but does play into effect, is hail damage. We've just been seeing increased intensity of the weather events. So, I think those are kind of the four that we've been seeing. [Participant I1]

Heat waves are very detrimental to canola production. A rise of 1.5°C lowered oilseed production by approximately 11.5%, causing an estimated yield loss of approximately 7.5% with a 1°C rise in air temperature (Wu et al. 2021). This reduction occurs due to the shortening of growing days, preventing biomass accumulation (Wu et al. 2021). Such impactful events cause a transition in the ecosystem, possibly decreasing biodiversity or dispersing insects to other regions, which was pointed out by a producer:

A warming climate is facilitating invasive species increasingly moving into canola production areas. Diseases, insects and weeds are and will all increase to some extent with a warming climate. So far, I see that as the biggest risk to canola profitability, if not production. [Participant S3]

So, besides adopting climate‐resilient genotypes, predicting climate variability and extreme event impacts is crucial to improve crop production (Raman et al. 2019). Flowering is the most sensitive stage of canola development to heat stress (Kirkegaard et al. 2021), followed by the pod‐filling stage (Raman et al. 2019). However, high‐intensity damage may also happen to seedlings (Wrucke et al. 2020) and compromise the whole growth cycle. Moreover, water availability to plant physiological processes can be decreased by heat stress, turning high temperatures even more problematic (Krishnan et al. 2011).

Floods are also recognized in the literature and to a moderate degree by producers in this study as harmful events to canola cultivation. Their damage varies in magnitude, spatial distribution, and duration. It causes cell saturation and root rotting by oxygen deficiency, stops nutrient transportation and availability, promotes nutrient deficiency or toxicity, and affects the soil microbiota. If the event lasts an extended period, it can lead to a complete crop loss. Besides, it causes soil erosion, waterlogging, nutrient leaching and runoff, and salinity, and reduces groundwater quality, resulting in a worse environment to cultivate crops with less nutrient availability (Fukao et al. 2019; Paul and Rasid 1993; Valipour 2014; Wetterauer and Killorn 1996). Perhaps more interesting is the low but consistent losses associated with rainstorms and the damage (ranging from low to severe) associated with hailstorms. The prairies are known for intense, short‐lived, spatially limited convective storms that can produce intense rain, large hail, high winds, and tornadoes (Stewart et al. 2019).

**FIGURE 3:**
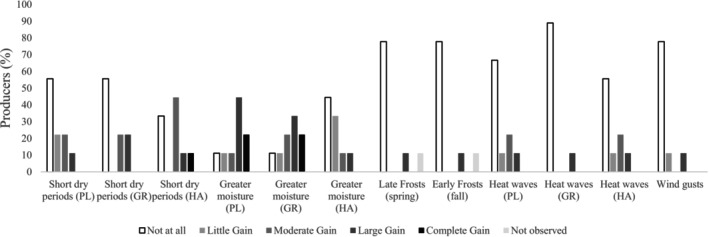
Percentage of participant producers who have experienced an improvement in canola production in growth and yield gain from extreme event occurrence at the beginning of the season (PL), during the growing season (GR), or the harvest season (HA).

However, when in a low or moderate state, causing greater water moisture, excess water may promote growth and yield gains during the whole season, especially in the planting period. This was reported by survey participants who have seen increases in production associated with increases in moisture (Figure 3). Optimum soil moisture improves nutrient availability and facilitates better nutrient uptake by the plant (Liyanage et al. 2022; Rahman et al. 2020). Heat waves were reportedly beneficial during the harvest season (Figure 3), which may help avoid mold infections (Jian et al. 2022) as the grains dry. Higher temperature amplitude is also expected to improve production since high temperatures at night may impact anthesis, yield, and seed components (Pokharel et al. 2020). As one producer surveyed declared:

Cool nights and warm days around 27°C are also beneficial pattern. Overcast clouds from forest fires (unfortunate) were a benefit from heavy sun that increased yield. [Participant S9]

Overall, canola yield (bushels per acre) has improved and increased in the last ten years, according to 90% of KIs. They attributed this improvement to better inputs and especially the availability of genetic advances:

Genetics are definitely a part of the solution to continuing to improve canola yields and, be able to grow in the environment that we're experiencing. But it's slow. So, its importance is high, but the impact does take time. So, it's up to seven to 10 years from the time that a new seed or variety can be available for sale once that breeding process is started. So, it's a long time before new genetics can make an impact, but there are improvements every year and, seed companies do invest a tremendous amount of money trying to bring the right genetics for farmers to be successful. [Participant I3]

These genetic advancements include pod‐shattering resistance, variety diversity that suits different regions, easy weed and pest control from herbicide and pesticide tolerance, the ability of canola to be grown in a wide‐ranging environment, water use efficiency, photosynthesis efficiency, disease resistance, abiotic stress resistance, and breeding for yield potential. Despite this, and the fact that canola is likely to be in high demand in the future for food oil and biofuel feedstock, one in three producers surveyed predict that their canola production will be similar to current levels five years from now. It should be noted that the remainder of producers do plan on increasing yield and profitability.

Developing greater tolerance to heat and drought is considered urgent in Canada (Kirkegaard et al. 2021). However, one KI questioned the focus of breeding programs, stating that the priority is to improve yield potential, leaving tolerance to abiotic conditions a low priority. This KI believes this is problematic because yield losses from hybrid canola cultivation due to flooding have been identified. Another KI questioned if focusing on genetic improvement is a sustainable approach since basic physiology means that there is a yield threshold that may be reached by breeders which could cause the yield to plateau. This yield potential threshold has been attained in diverse countries, and many studies have researched the limiting factors to achieve this potential on crop production worldwide (Page et al. 2021; Reynolds et al. 2022; Tian et al. 2021; Yaqub et al. 2022). Yet breeding is not the only factor involved in yield response. The interaction between genotype and environmental conditions linked to management practices determines canola yield (Ahmad et al. 2023), which corroborates the perspective of one KI: “The genetics are always trying to improve the yield. So, I think the yield is increasing. But climate change might limit what we can do.” [Participant I9]

While canola yields were seen to have been increasing, participants stated that canola productivity (profitability) has worsened in the last 10 years, primarily due to the necessary investment to cultivate it. Since the release of canola hybrids, the higher cost of seed has become a disadvantage. One KI mentioned that “the cost of canola has increased 40% in the last five years only” [Participant I4]. For example, a registered herbicide‐tolerant canola seed cost was $695 per bag in January 2020, spiking to $845 in January 2024 (Alberta Agriculture and Irrigation 2024). Canola seed sales are currently showing daily fluctuation costs in the Prairie Provinces by approximately C$13 per tonne (PDQ 2024). Still, 78% of the participant producers see using tolerant seeds as a viable adaptation strategy:

As we see our canola seed escalate from $600 to $800 a bag. It starts to get you to question how much you want to put into genetics. But if the returns are there, I could see an amount going towards genetics for sure. [Participant I6]

**FIGURE 4:**
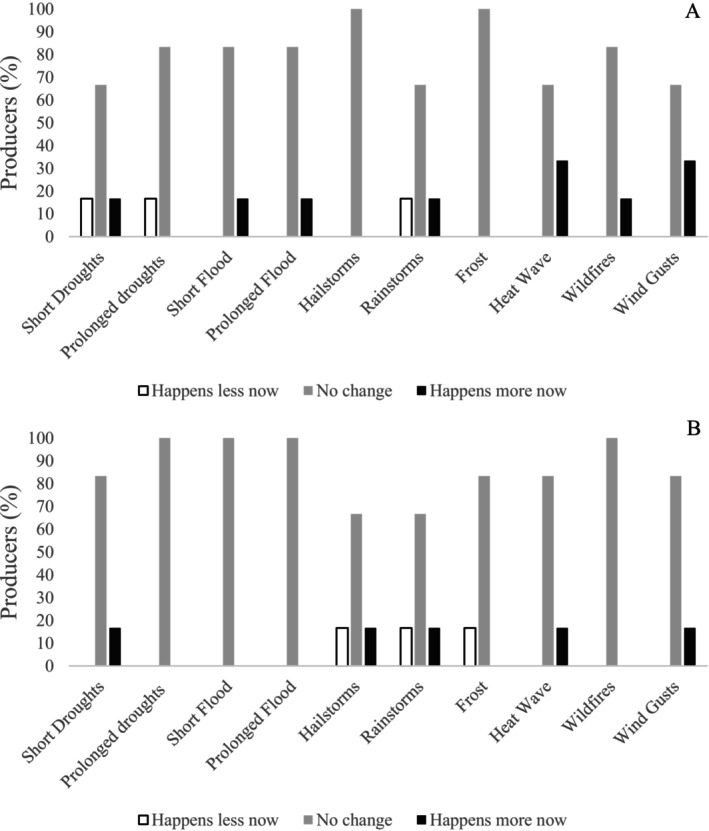
Percentage of participant producers who have perceived less, no change, or more extreme events in the last 30 years during the planting season (A) and the harvest season (B). *n* = 9.

When looking at changes over time, respondents did not really identify many changes either in the planting season (4A) or harvesting (4B). There were some indications that heat waves and wind gusts happen more now than 30 years ago during the planting season, but this was not identified by many participants (Figure 4A). Heat stress can harm canola, reducing seed production, oil content and weight loss, and yield components (Koscielny and Duncan 2024; Lohani et al. 2022). From a one‐week heat wave, effects can last weeks to months after (White et al. 2023), impacting canola's floral development and yield (Rivelli et al. 2023). Wind gusts contribute to spreading and redirecting wildfires (Cruz 2022), reduce pollination by bees (Farooqi et al. 2021), carry particles (such as sand) capable of wounding canola seedlings (Huang et al. 2024), and alter the occurrence and impact of pests and diseases (Huang et al. 2024; Wadhwa and Malik 2024). Regarding other changes over time, none of the producers expressed changes in the frequency of prolonged droughts, floods and wildfires during the harvest season in the last 30 years (Figure 4). Further, perceptions of climate change were not influenced by level of education, age, or gender, confirming findings of a study by Foguesatto and Machado (2021). Conversely, approximately 60% of KIs remarked seeing more change in climate and 30% observed uncertainty. Similar to producer responses, 10% reported less change since the 1980s.

### 3.2 Climate‐Smart Farms

All KIs agreed that producers know and understand climate variability and resilience, use their experience, and evaluate their reality before implementing new strategies. The following quote summarizes the collective thought:

Growers across Western Canada are not uninformed, or they've got a lot of experience of growing canola in many different growing season conditions. So, the expectation for growers is to be cautious and to be conservative, for the most part, because of the risk or potential of being profitable or not. So, I think right now producers are going to continue to do what they've always done. Which is making decisions based on what they've got for information ahead of them. And understanding or evaluating the inventory of what they've got for their land and the resiliency that they've been able to build into it. [Participant I3]

Still, 60% of the total participant producers declared that they had never received public financial emergency support for impacts related to extreme events, such as emergency support for flooding, drought, animal losses, etc. Besides, 23% of the total participants who have already had an insurance plan have abandoned it.

Participant producers who indicated a lack of concern over how their farm practices must adapt to a changing climate indicated that they use zero or only one source of information (agronomist or animal scientist) to know more about the changing climate. Producers who declared being moderately concerned use five or six sources of information, and producers who are extremely concerned use at least ten sources of information, which includes local news, newspapers, social media, other farmers, growers' associations, agronomists or animal scientists, universities and other educational institutions, research institutions, conferences, scientific publications, agricultural fairs, and governmental agencies.

With respect to decision‐making authority, 62% of the canola producers indicated that their decision‐making and control over the farm is fully independent. The 38% that indicated semi‐dependent control related this semi‐dependency to operational or financial constraints or co‐management amongst shareholders (Table 2). Even though the study resulted in a small sample size, we found high correlations with low *p*‐values between participant concern about adapting to a changing climate and how much specific climatic events (flood, drought, frost, heatwave) have affected their canola production (Table 3). Hailstorms, storms, wildfires, and wind gusts were not correlated, and results are not shown.

**TABLE 3:** Correlation and *p*‐value between concern about adaptation and events faced by the producers (*n* = 9).

Concern	Drought (short period)	Drought (prolonged period)	Flood (short period)	Flood (prolonged period)	Frost	Heat wave
Correlation	0.86	0.69	0.75	0.79	0.79	0.75
*p*‐value	0.003	0.041	0.019	0.012	0.011	0.021

**FIGURE 5:**
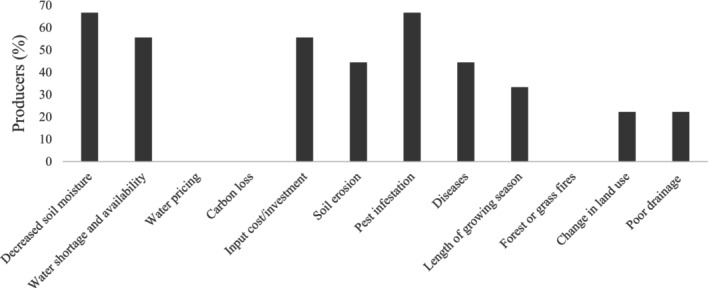
Percentage of participant producers who have faced these conditions and adapted their canola production.

**FIGURE 6:**
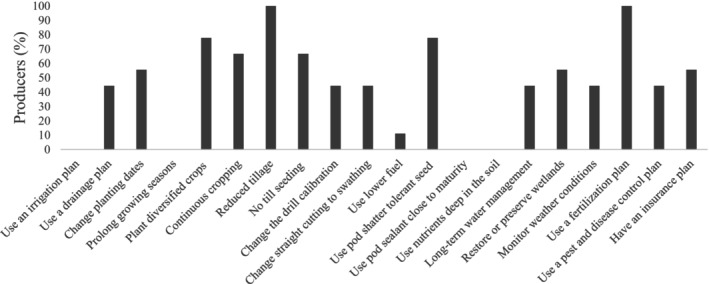
Percentage of participant producers who consider the adaptation strategies viable.

Approximately 70% of the participant producers have faced and adapted their production to decreased soil moisture and pest infestations, whereas 55% faced and adapted to input cost and water shortages (Figure 5). Improving the water use efficiency of canola is crucial to avoid constraints on production (Liyanage et al. 2022). One adaptation strategy that may maintain water moisture in the soil is reduced or no tillage. 90% of participant producers implement reduced tillage, and all respondents think it is a viable adaptation strategy (Figure 6). One KI declared that they also implement this strategy: *“*We're doing no tillage and things like that to try and preserve some of the moisture on the dry years” [Participant I6]. Reduced tillage promotes sustainable cropping systems by reducing soil erosion (faced by 45% of the producers), incorporating residue, and improving the physical characteristics of the soil and nutrient levels (Mayer et al. 2021).

Of all adaptation strategies identified as viable (Figure 6), fertilization plans and reduced tillage were identified most frequently, followed by crop diversification and pod shatter tolerant seeds. Genetic adaptations, such as seeds tolerant to pod shatter, were referenced more than 60 times in KI interviews in addition to almost 80% of producers surveyed. The speed of genetic development for canola is seen as a considerable benefit. In fact, genetic improvement and breeding can improve tolerance to abiotic stresses, herbicides and pesticides, water and nitrogen use efficiency, seed content, and yield potential (Batool et al. 2023; Deng et al. 2023; Nelson et al. 2022; Raman et al. 2022; Sharma et al. 2024). More generally, KIs cited water management and crop rotation (67% each), reduced or no tillage (56%), fertilization and weed control (45%), and seeding (34%) as viable adaptation strategies. Of the limited producers surveyed, 40% identified drainage as a viable strategy, but no one identified irrigation as viable, possibly due to geographic location. Early (spring) and late (fall) frost can be avoided by changing sowing dates (Secchi et al. 2021), which almost 80% of the participant producers have already implemented as an adaptation strategy.

Hence, awareness of climate variability and extreme events is crucial to developing and expanding adaptation strategies (van Tilburg and Hudson 2022). Still, even with this present variability, the adaptation strategies already implemented are operating well, as mentioned by another producer, “So far, and as far as we can predict, our strategies are helping us adapt quite well.” [Participant S3]

Most participant producers claim that internal financial resources are a major barrier when implementing a new adaptation strategy. They have also stopped or abandoned a strategy because of financial constraints, or because the strategy failed to perform as expected. One KI noted that financial support from the government is a determinant of adaptation at the farm level:

Well, I don't think, on average, we have too many acres in this province that are protected with either drought or flood tile drainage. So, I think we have a long way to go to increase those acres. It's such a significant cost to implement irrigation when 9 out of 10 years we are getting enough moisture through the environment itself. And, on the flip side, if we have one‐in‐ten‐year floods, do you want to invest that $1000 an acre on tile drainage? So, those are the questions we battle and if there was funding, I think I'd make the question of whether it should be done a lot easier. If there was some type of government assistance to help with that… So, how do we get producers to adapt and change when they know that it's an issue? But the funds aren't there currently just due to increased land costs, interest rates and everything else that comes along with production. There just might not be that extra return available to put back into creating these long‐term things. The problem is we don't have an idea that we're only going to go drier. If we're only going to have a drier environment, we could focus on irrigation. But we're having flip flops in two to three years. So, there's such extreme events that we have to take care of both events. And that is compounded the cost. [Participant I6]

Finance is one of the most common constraints to climate change adaptation globally and highly relates to implementing new technology and infrastructure (Thomas et al. 2021). Technological advances have allowed Canadian farms to double their size since 1980, and the sector is expected to keep growing as Canada is rich in land and water resources (Agriculture and Agri‐Food Canada 2022). However, there are programs that have been established to fund climate resilience. The federal government started the Agricultural Climate Solutions program, which funds climate change solutions to help farmers with the On‐Farm Climate Actions Fund (Agriculture and Agri‐Food Canada 2021a). They also promote a collaborative approach with farmers, scientists and shareholders to test and implement sustainable on‐farm management practices focused on climate change mitigation with Living Labs (Canada 2023b). Alberta has six grant programs at the provincial level focused on farmers within the Sustainable Canadian Agricultural Partnership to support producers to improve, expand, conserve, and adopt sustainable management practices (Government of Alberta 2024c). Manitoba has four programs focused on climate change and the environment (Government of Manitoba 2024a). Saskatchewan has 11 programs in partnership with the federal government, with the most significant difference being the irrigation program (Government of Saskatchewan 2024d). Although 45% of the KI agreed that the governmental funding clusters have focused on climate‐smart farming solutions, they recognized that, lately, the clusters have presented strict criterion for what can be funded:

The last funding cluster was really hard because they only wanted to farm or to fund kind of environmental‐impact‐type work. Which they had really strict criteria on what that was. And I think the lack of understanding on the government side of things about how general practices and improving productivity on farm have the impact of increasing and making them more climate‐smart farms was kind of lost in translation there. They really wanted projects that you're directly measuring to low emissions or that you're looking specifically at cover crops. But there's a lot of other things that farmers are doing or can be doing. So, they did put a big focus on climate‐smart solutions, but they weren't doing it in the right way, and they were really excluding some factors or some research topics that could have provided a lot of value but were excluded in their type of funding because of some really strict criteria and kind of narrow thinking. All their funding was credit lower compared to what has been passed, so it has been a downward trend of government funding in general, whether it includes in climate solutions or not. [Participant I4]

The Canola Agronomic Research Program (CARP) from the Canola Council of Canada is funded by the Alberta Canola, Manitoba Canola Growers and SaskCanola. In four of the last five years, 80% of CARP‐funded projects focused on pests and diseases. Funding in 2022 was more diverse and included heat and drought tolerance. Diversity in research is highly needed to achieve climate change adaptation and mitigation (Neta 2023).

## 4 Final Considerations and Future Implications

### 4.1 Outreach

When considering climate adaptation on farms, it is important to make adequate, relevant information available to producers (Kamruzzaman et al. 2023). Although KIs indicated that all research findings are published and available on websites, e.g., *“*We have the 17 years of research results at first hand if they need them right on the website.” [Participant I1], this did not align with survey responses from producers, who identified access to information as a barrier to adaptation. KIs also referred to regular newsletters, responding to emails, *one‐on‐one* telephone conversations, and *in‐person* conversations at extension events and workshops as mechanisms through which they share their expertise and knowledge. However, survey participants were still looking for “Access to qualified on‐farm testing and experiences. Access to proactive government consultants regarding these matters.” [Participant S9]

Despite this disconnect, *communicate* was one of the most cited words from KI transcripts. For example, research scientists were primarily concerned with communicating their results:

If we do have data or do have knowledge on how the conditions might be affecting something, we're concerned about trying to communicate that and make sure that knowledge or information is out there for producers. That's kind of our biggest role as research scientists. It is either conducting research to help try and answer some of those questions or help improve the preparedness of our industry for extreme events. But also, then communicating what we do know and trying to manage our research trials in accordance, as well. [Participant I8]

Most KIs agreed that the effectiveness of different communication techniques changes over time. Even though research results and annual reports are available on websites, social media was identified as necessary to reach producers, especially the younger generations: *“…*in the social media nowadays, even the young, even the old farmers. I think like the visuals will be more appealing, I would say” [Participant I5]. *However, it was also identif*ied as a time‐consuming activity that requires skilled staff.

Producers also hold relevant information from years of farming experience: “Our farm has been here for more than 100 years. We've seen every cycle from dry to very wet, and both within the past twenty years.” [Participant S10] Hence, collaborative initiatives such as participatory research to integrate knowledge by combining information learned through generations of producers and Traditional Ecological Knowledge can create a more focused decision‐making process to serve local needs (Kröbel et al. 2021).

### 4.2 Public Policies and Research

Currently, the Canadian government has canola policies focused on accessibility to canola products in the market, canola exports and international trade, sustainable practices at the field level, and research (Canada 2021b, 2024b; Canola Council of Canada 2024a, 2024b). However, participants identified the need for new or improved public policies, research, and funding regarding:

- The canola industry and seed markets

As canola growth becomes a lucrative business, it is essential to develop a public policy about company trials and the monopoly of the seed market. This monopoly has been discussed in the academic literature by Lindquist et al. (2022) and mentioned by one of the KI:

It's getting to the point now between companies that they don't want to run variety trials publicly anymore because of the attempt possibly to monopolize the seed market and so, there needs to be a bit of more public resolution on that level. [Participant I1]

b Diversification in agriculture

There is a need to develop and fund research to support diversification processes and better agronomic practices for new crops on the Prairies and to improve the growing process for already established crops, as diversification can be beneficial to crops (Blade and Slinkard 2022; Connor 2001; Masse et al. 2025). This demand was indicated by one KI: “One public policy I can think of is creating organizations…which are designed to broaden the diversification approaches to things.” [Participant I2]

c Land and water management by producers.

There is a need to establish more viable regulations or processes around water management and soil aeration. One participant described inter‐provincial differences in approval processes for tile drainage projects, for example, which can include permission from neighbors (Melchior 2017). Another suggestion was the use of on‐farm reservoirs to store water during rainy days and use this water to irrigate the crops when necessary: “I think we don't have many farms or maybe there are only few in [province] where people store water at the farm and then they use when they need to.” [Participant I5]. This practice benefits water management at the field level (Tiessen et al. 2011) but needs to consider up‐to‐date information to promote suitable and timely adaptation to changes in demand (Garcia et al. 2020).

d Climate‐smart farms

More information and data from pilot‐level testing on climate‐smart activities must be shared with producers to build confidence in adaptation and climate change mitigation. Enhanced understanding of potential long‐term trends in local diseases is a critical element in this regard

e Precision Agriculture, Artificial Intelligence (AI), and Genetics

Appropriate genetic strains for changing environmental conditions are a critical adaptation strategy. Precision technology in agriculture, such as the Clustered Regularly Interspaced Short Palindromic Repeats (CRISPR) used to modify the DNA and genetically improve canola, must be explored but also regulated further: “There's potential to have to regulate CRISPR technology because this technology is almost so easy to do.” [Participant I1] Gene‐editing technology is not unknown to the government and regulatory entities. Health Canada released a scientific note stating that this technology does not raise concerns regarding food safety and should be regulated within the Novel Food Regulations as with any other plant breeding technology (Health Canada 2024). Gene‐edited crops have also been identified as safe for livestock feed since June 2024 (Government of Canada 2024).

f Sustainable fertilization

Given high use of phosphorus and potash, there is a need to avoid a decline in nutrient sources over time that could prevent sustainable crop growth. Associated with this is regulation of nitrification inhibitors and reduction in use of greenhouse gas fertilizers, such as synthetic nitrogen fertilizers based in ammonia that emit CO_2_, N_2_O, and CH_4_ (Gao and Cabrera Serrenho 2023). In 2021, the Minister of Agriculture and Agri‐Food of Canada announced an investment in pulse crops aiming to encourage farmers to include and rotate nitrogen‐fixing crops to decrease the use of chemical nitrogen fertilizers (Canada 2021b). Governments in all three Prairie provinces provide information on nitrogen and phosphorus fertilization on websites and offer additional help (Government of Alberta 2024a, 2024b; Government of Manitoba 2024c, 2024b; Government of Saskatchewan 2024a, 2024b, 2024c), but given the potential communication disconnects identified in this study, other avenues to communicate information could be explored.

g Hail and crop insurance

Five KIs mentioned crop insurance during the interviews. One KI recommended building climate‐smart incentives into insurance programs. Farmers who implement specific practices to reduce the risk of losses due to climate variability would be given a better deal on crop insurance premiums. There is also a need for the federal government to fund risk management tools. According to the federal government, crop insurance programs in Saskatchewan are well designed to respond to weather‐related production losses and support crop producers with lower costs and improved coverage (Agriculture and Agri‐Food Canada 2024a).

### 4.3 Future Generations of Producers

In response to a question regarding advice for young producers and youth who might plan to produce canola in the future, recommendations were multi‐fold. First, to be as informed and as up to date on technology and holistic approaches to farming as possible. Second, to listen to older generations and peers, to be open to change, and to plan for and not be discouraged by bad years – “be flexible financially to always have reserves to make it through a bad season” [Participant S3]. Third, to take advantage of crop insurance and programs that support young farmers and to maintain contact with specialists for additional recommendations and beneficial opportunities. Finally, to prioritize work‐life balance and mental health.

## 5 Conclusions

This study provides a small snapshot of current and potential canola production on the Prairies within the context of a variable and changing climate. Changes in soil moisture and drought were identified as the biggest concern for canola production on the Prairies. However, producers respond to uncertainty and change by taking precautions and analyzing economic impacts of different on‐farm investments, whether implementing new adaptation strategies, purchasing new equipment, or collaborating with research and policymakers. Genetic improvement and breeding were the top adaptation strategies identified. While pursuit of profitable production is a driver of decision‐making, many identified that investing in yield‐increasing strategies is not the only factor in achieving sustainable growth. Communication between producers, specialists, and researchers is important for responding to producer concerns and for improving the preparedness of the whole industry for extreme events. Arguably, a sustainable approach is crucial to securing the farm's future for next generations. In addition, it is necessary to continue researching climate‐smart solutions, connecting producers and the industry more broadly with universities and research institutes, as well as developing sector‐specific regulations that help producers adapt and continue cultivating crops essential for society. In turn, this requires long‐term investments in human, financial, and program resources, especially at federal and provincial levels.

## Funding

This work was supported by the Natural Sciences and Engineering Research Council of Canada, NSERC Discovery (Grant RGPIN‐2019‐04590), NSERC Discovery (Grant RGPIN‐2019‐06894).

## Ethics Statement

Ethical approval for this study was obtained from the University of Saskatchewan Behavioral Research Ethics Board (certificate # BEH 4413).

## Conflicts of Interest

The authors declare no conflicts of interest.

## Supporting information


**Table S1:** Aggregated data gathered through the online survey (*n* = 13).
**Table S2:** Aggregated data gathered through the key informant interviews (*n* = 9).

## Data Availability

Data underlying the findings of this study are available in the Supporting Information file. Due to privacy and ethical constraints, data are aggregated.
