# Supplementary material for: Initial Exploration of Canola Producers' Approaches in Response to Changing Climate in the Canadian Prairie Provinces
Source: Plant Environ Interact. 2026 Jan 4;7(1):e70107. doi: 10.1002/pei3.70107 (PMC12766075; doi:10.1002/pei3.70107)
Supplement: Supplementary file 1 — Table S1: Aggregated data gathered through the online survey (n = 13). Table S2: Aggregated data gathered through the key informant interviews (n = 9). [file PEI3-7-e70107-s001.pdf]

Plant-Environment Interactions Journal

Supplementary Information for

Initial exploration of canola producers' approaches in response to changing climate in the Canadian Prairie Provinces

Yohanne Larissa Gavasso-Rita[1,2*], Simon Michael Papalexiou[3,1,2,4], Yanping Li[5], Amin Elshorbagy[1,2], Corinne Schuster-Wallace[6,2]

[1]Department of Civil, Geological, and Environmental Engineering, University of Saskatchewan, Canada; [2]Global Institute for Water Security, University of Saskatchewan, Canada; [3]Institute of Global Water Security, Hamburg University of Technology, Hamburg, Germany; [4]Schulich School of Engineering, University of Calgary, Canada; [5]Department of Physics and Astronomy, Western University, Canada; [6]Department of Geography and Planning, University of Saskatchewan, Canada.

The following questions were asked during the key informant interviews.

1. Please, tell me about your role at [*organization*] and your work there regarding extreme events and agriculture.
2. Overall, in your opinion, has canola's productivity improved or worsened in the last ten years?
    a. What are the factors responsible for this improvement or loss?
3. How are extreme weather events currently affecting the food and energy sectors?
    a. More specifically, how are those events affecting canola production?
4. Which extreme events worry you most when thinking of the future of canola production?
    a. What data can you provide to support the producers before and during the growing season?
    b. What is usually the producers' first response when [*cited events*] are forecasted to happen in the long term?
    c. Do you think this response is adequate? If not, what do you believe this response should be?
    d. How would you rate the producers' and the market's preparedness if production is negatively affected?
    e. How does your organization deal with a severe situation?
5. What is your perception of climate-smart farms in your province?
    a. What are the current gaps that need improvement?
    b. How's the Agriculture sector/association/governmental investment on climate-smart farms?
6. What is the most important agricultural adaptation strategy in this province? Why?
    a. How do producers react when your organization suggests a new adaptation plan?
    b. How does your organization communicate this need?
7. How important is the role of genetics in adaptation strategies?
    a. How are genetics seen by the producers? A big expense, a necessary investment, etc.?
8. What are your thoughts on public policies regarding canola production losses due to extreme events?
9. What would you like to see change?
    a. What would need to happen for this to occur?

10. Where do you expect the canola sector to be in five years from now?

11. Within the effects of extreme events on provincial canola production, what advice do you have for youth who plan on being future producers?

12. Is there anything else you'd like to share with me that we haven't covered?

The following questions were used as the survey instrument.

Screening Questions * indicates a required answer.

1. Are you at least 18 years of age? *
   - Yes
   - No *Skip to section 2 (Thank you for your time!)*

2. Have you produced canola for at least one growing season? *
   - Yes
   - No *Skip to section 2 (Thank you for your time!)*

3. Is your farm located in the Canadian Prairie Provinces (Alberta, Manitoba, Saskatchewan? *
   - Yes
   - No *Skip to section 2 (Thank you for your time!)*

4. By completing and submitting the questionnaire, **YOUR FREE AND INFORMED CONSENT IS IMPLIED** and indicates that you understand the above conditions of participation in this study. Do you wish to continue? *
   - Yes
   - No *Skip to section 2 (Thank you for your time!)*

If 'No' is the answer to any of the screening questions, the survey ended, not giving access to the questionnaire.

Thank you for your time!

We value your well-being and appreciate your participation!

The questionnaire proceeds if all the screening questions are answered with 'Yes':

General Information about your farm

5. What size is your farm?

- ○ 1 - 500 acres
- ○ 501 - 1000 acres
- ○ 1001 - 2000 acres
- ○ 2001 - 3000 acres
- ○ 3001 - 4000 acres
- ○ 4001 - 5000 acres
- ○ 5001 - 6000 acres
- ○ 6001 - 7000 acres
- ○ 7001 - 8000 acres
- ○ 8001 or more acres

6. How long have you been farming?

- ○ Less than 5 years
- ○ 5 - 15 years
- ○ More than 15 years

7. How long have you produced canola?

- ○ 1 -3 years
- ○ 4 -6 years
- ○ 7 - 9 years
- ○ More than 9 years
- ○ I don't know
- ○ Prefer not to say

8. Please, provide the FIRST FIVE characters of your postal code: ___________

9. What is your most profitable farming activity? *[Please select one]*

- ○ Oilseeds
- ○ Grains
- ○ Pulses

- Other crops
- Vegetables, fruit, or nuts
- Greenhouse, nursery, or floriculture
- Beef cattle ranching
- Dairy cattle and milk production
- Poultry and egg production
- Pig / Hog
- Sheep / goat
- Other (please specify): _________

10. What is the scope of your commercial activity? *[Choose all that apply]*

- Within the Municipality
- Within the Province
- Within the Prairies
- National
- International
- Not commercial

11. Is Canola production a primary source of farm income?

- Yes
- No
- Prefer not to say

12. Is canola regularly a top economic performer on your farm?

- Yes
- No
- Prefer not to say

13. What is your farm structure? *[Please select one]*

- Sole proprietorship (the farm is operated by a single individual/family)
- Partnership (two or more people/families operate the farm)
- Corporation (the farm is operated by a distinct legal entity)
- Prefer not to say
- Other (please specify): __________

14. How many employees do you have?

- 0
- 1 - 2
- 3 - 4
- 5 - 6
- 7 - 8
- 9 – 10
- More than 10
- Other (please specify if full time paid, seasonal paid, family members paid, or family members unpaid): __________

15. What degree of decision-making and control do you have over this farm?

- Fully independent
- Semi-dependent – financial constraints
- Semi-dependent – operational constraints
- Fully dependent (e.g. decision comes from management)
- Prefer not to say
- Semi-dependent – other constraints (please provide): ___________

16. Has your farm business received public financial support (e.g. emergency support for flooding, drought, animal losses, etc.)?

- Yes
- No
- I don't know
- Prefer not to say

Experiences with a Changing Climate

17. Are you concerned about how your farm practices must adapt to a changing climate?

- Not at all
- A little
- Moderately
- Quite a bit
- Extremely

18. What sources of information do you use to know more about the changing climate? *[Choose all that apply]*

- Local News
- Newspaper
- Social Media (e.g. Facebook, Twitter, Instagram, Snapchat...)
- Other farmers
- Suppliers
- Growers Associations
- Agronomist/Animal Scientist
- Family or friends
- Universities and other educational institutions
- Research Institutes
- Conferences, meetings, scientific publications
- Fairs or product shows
- Governmental agencies
- Other (please specify): __________

19. Have you experienced any of these in the last 10 years during the PLANTING season?

- Droughts (short period)
- Prolonged droughts (more than six months)
- Flooding (short period, e..g. water logging)
- Prolonged flooding (more than a week)
- Hailstorms
- Storms
- Frosts
- Heat waves
- Wildfires
- Wind gusts

20. Have you experienced any of these in the last 10 years during the HARVEST season?

- Droughts (short period)
- Prolonged droughts (more than six months)
- Flooding (short period, e..g. water logging)
- Prolonged flooding (more than a week)

- Hailstorms
- Storms
- Frosts
- Heat waves
- Wildfires
- Wind gusts

21. Have you seen changes in these over the last 30 years during the PLANTING season? [M*ark only one per row]*

	Happens less now	No change	Happens more now
Droughts (short period)			
Prolonged droughts (more than six months)			
Flooding (short period, e.g. water logging)			
Prolonged flooding (more than a week)			
Hailstorms			
Storms			
Frosts			
Heat waves			
Wildfires			
Wind gusts			

22. Have you seen changes in these over the last 30 years during the HARVEST season? [M*ark only one per row]*

	Happens less now	No change	Happens more now
Droughts (short period)			
Prolonged droughts (more than six months)			
Flooding (short period, e.g. water logging)			
Prolonged flooding (more than a week)			
Hailstorms			
Storms			
Frosts			
Heat waves			
Wildfires			
Wind gusts			

23. How much have these events affected your canola production in growth and yield losses? [*Mark only one per row]*

	Not at all	Little Loss	Moderate Loss	Large Loss	Complete Loss	Not observed
Droughts (short period)						
Prolonged droughts (more than six months)						
Flooding (short period, e.g. water logging)						
Prolonged flooding (more than a week)						
Hailstorms						
Storms						
Frosts						
Heat waves						
Wildfires						
Wind gusts						

24. Have you had to submit an insurance claim due to losses caused by any of these extreme and high-impact events?

- Yes
- No
- Prefer not to say

25. How much has changing climate events improved your canola production in growth and yield gains? [*Mark only one per row]*

	Not at all	Little Gain	Moderate Gain	Large Gain	Complete Gain	Not Observed
Short dry periods (1-2 weeks) at the beginning of the growing season						
Short dry periods (1-2 weeks) during the growing season						
Short dry periods (1-2 weeks) at harvest						
Greater moisture availability at the beginning of the season						
Greater moisture availability during the growing season						

Greater moisture availability at harvest	◯	◯	◯	◯	◯	◯
Late Frosts (spring)	◯	◯	◯	◯	◯	◯
Early Frosts (fall)	◯	◯	◯	◯	◯	◯
Heat waves at the beginning of the season	◯	◯	◯	◯	◯	◯
Heat waves during the growing season	◯	◯	◯	◯	◯	◯
Heat waves at harvest	◯	◯	◯	◯	◯	◯
Wind gusts	◯	◯	◯	◯	◯	◯

26. Is there anything else you would like to share about the changing climate?

____

Climate-Smart Farming

27. What conditions have you faced and adapted your canola production for? *[Choose all that apply]*

- ◯ Decreased soil moisture
- ◯ Water shortage and availability
- ◯ Water pricing
- ◯ Carbon loss
- ◯ Input cost/investment
- ◯ Soil erosion
- ◯ Pest infestation
- ◯ Diseases
- ◯ Length of growing season
- ◯ Forest or grass fires
- ◯ Change in land use (e.g. farming to ranching)
- ◯ Poor drainage (e.g. water logging)
- ◯ Other (please specify): _________

28. Which of the following do you consider to be viable adaptation strategies? *[Choose all that apply]*

- ◯ Use an irrigation plan
- ◯ Use a drainage plan

- Change planting dates
- Plant diversified crops (e.g. crop rotation, intercropping, mixed cropping, cover crop)
- Continuous cropping
- Reduced tillage
- No till seeding (e.g. use less fuel)
- Change the drill calibration
- Change straight cutting canola to swathing
- Use pod shatter tolerant seed varieties
- Use pod sealant close to maturity
- Use nutrients deep in the soil
- Long-term water management
- Restore or preserve wetlands
- Monitor weather conditions
- Use a fertilization plan
- Use a pest and disease control plan
- Have an insurance plan
- Other (please specify): ___________

29. Which of the following strategies have you implemented or abandoned?

	Never used it	Tested	Implemented	Abandoned	Not aware
Use an irrigation plan					
Use a drainage plan					
Change planting dates					
Plant diversified crops (e.g. Crop rotation, intercropping, mixed cropping, cover crop)					
Continuous cropping					
Reduced tillage					
No till seeding (e.g. Use less fuel)					
Change the drill calibration					
Change straight cutting canola to swathing					
Use pod shatter tolerant seed varieties					
Use pod sealant close to maturity					
Use nutrients deep in the soil					
Long-term water management					
Monitor weather conditions					
Restore or preserve wetlands					
Use a fertilization plan					

Use a pest and disease control plan	◯	◯	◯	◯	◯
Have an insurance plan	◯	◯	◯	◯	◯

30. If you stopped or abandoned a strategy, please tell us why. *[Choose all that apply]*

◯ Cost/investment
◯ Work overload
◯ The strategy failed to perform
◯ Difficulty
◯ Worker shortage
◯ Uncertainty of regulatory approval
◯ Water shortage
◯ I don't know
◯ Prefer not to say
◯ Other (please specify): _________

31. What are the barriers or drivers you face when implementing an adaptation strategy? *[Choose all that apply]*

	Major barrier	Minor barrier	Neither a barrier nor a driver	Minor driver	Major driver
Strategic goals/culture	◯	◯	◯	◯	◯
Internal financial resources	◯	◯	◯	◯	◯
Qualified personnel	◯	◯	◯	◯	◯
Information about the new product or technology	◯	◯	◯	◯	◯
Information about market and profit	◯	◯	◯	◯	◯
Regulations (e.g. environmental tax)	◯	◯	◯	◯	◯
Cost/investment	◯	◯	◯	◯	◯
Access to bank loans	◯	◯	◯	◯	◯
Access to governmental financial support	◯	◯	◯	◯	◯
Access to appropriate infrastructure	◯	◯	◯	◯	◯
Other (please describe): _________					

32. Is there anything else you would like to share about your strategies to adapt and avoid losses due to extreme weather?

____

Socio-economic and Demographic Information

33. How do you identify?

- Man
- Woman
- Gender-fluid, Non-binary and/or Two-spirit
- Prefer not to say

34. How old are you?

- 18 – 29
- 30 - 39
- 40 - 49
- 50 - 59
- 60 - 69
- 70 - 79
- 80 or more

35. How long have you lived or worked on this farm?

- 1 - 3 years
- 4 - 6 years
- 7 - 9 years
- More than 9 years

36. What education experience do you have? *[Choose all that apply]*

- Less than high school diploma or its equivalent
- High school diploma or a high school equivalency certificate
- Trades certificate or diploma
- College or other non-university certificate or diploma (other than trades)
- University certificate or diploma at graduate level (Bachelor's)
- University certificate or diploma at postgraduate level (Master's, PhD, Professional Degree)
- Land-based learning or traditional knowledge

37. What is your yearly income from farming before taxes?

- Less than $25,000
- From $25,000 to $50,000
- From $50,001 to $75,000

- From $75,001 to 100,000
- From $100,001 to 150,000
- From $150,001 to $200,000
- From $200,001 to $250,000
- From $250,001 to 300,000
- From $300,001 to $350,000
- From $350,001 to $400,000
- More than $400,000
- Prefer not to say

38. How do you see your canola production in five years from now?

- Significantly increasing production *Follow up question 36*
- Likely producing more *Follow up question 36*
- Similar production levels
- Likely producing less *Follow up question 37*
- Stopping production completely *Follow up question 38*
- I don't know

39. How do you plan to achieve this expansion?

40. Why do you think your production will decline?

41. Why will you stop production?

Thank you for your participation!

Please, press SUBMIT to complete the study.

We highly appreciate your time and participation in this survey!

Table S1. Aggregated data gathered through the online survey (n=13).

		Age (years)						Gender identification			Years producing canola				I don't know	No/Not observed
		18-29	30-39	40-49	50-59	60-69	No response	Man	Woman	No response	1-3	4-6	7-9	>9		
Concern about adapting farm practices	Not at all				2			1		1		1		1		
	A little	1						1				1				
	Moderate	1			1			1	1					2		
	Quite a bit		1		1	1		2	1					3		
	Extremely			1				1						1		
	No response						4			4	1	1		2		
Source of information	Local news	1		1				2				1		1		
	Newspaper	1		1	1			3						3		
	Social media		1	1	1			1	2					3		
	Other farmers			1				1						1		
	Suppliers															
	Growers Associations	1	1	1				2	1					3		
	Agronomist / Animal Scientist	1	1	1	2			2	2	1		1		4		
	Family or friends															
	Universities and other educational institutions		1	1	1	1		2	2					4		
	Research institutes	1	1	1	2	1		4	2					6		
	Conferences, meetings, scientific publications	2	1	1	2				2			1		5		

	Fairs or product shows	2	1	1	2			4	2			1	5		
	Governmental agencies		1	1	1	1		2	2				4		
	None				1			1					1		
	No response						4			4	1	1	2		
Experienced in the last 10 years during planting season	Droughts	2	1	1	1			4	1			1	4	2	
	Prolonged droughts	2						2				1	1	1	
	Flooding	2	1	1	1			4	1			1	4	1	
	Prolonged flooding	1	1	1				2	1			1	2	1	
	Hailstorms		1	1	2			3	1				4		
	Storms	2	1	1	1			4	1			1	4		
	Frosts	2	1	1	3	1		6	2	1		2	7		
	Heat waves	2	1	1	1			4	1			1	4	1	
	Wildfires				1			1		1		1			
	Wind gusts	2	1	1	1			4	1			1	4		
	No response						2			4	1	1	2		
Experienced in the last 10 years during harvest season	Droughts	2	1	1	1			4				1	4		
	Prolonged droughts	1						1				1			
	Flooding		1	1				1	1				2		
	Prolonged flooding	1	1					1	1				2		
	Hailstorms	2	1	1	1			4	1			1	4		
	Storms	2	1	1	1			4	1			1	3		
	Frosts	1	1	1	1			2	1	1		1	3		
	Heat waves	2	1	1	1			4	1			1	4		
	Wildfires														
	Wind gusts	2	1	1	1			4	1			1	4		
	No response				1	1			1	4	1	1	3		
Seen changes in the last 30 years during planting season	Droughts	1		1				2					2		4
	Prolonged droughts	1						1					1		4
	Flooding				1			1					1		5
	Prolonged flooding				1			1					1		5
	Hailstorms														6
	Storms			1	1			2					2		4
	Frosts														6
	Heat waves	1		1				2					2		4
	Wildfires	1						1					1		5

	Wind gusts	1			1			2					2		4
	No response		1		1	1	4		2	4					
Seen changes in the last 30 years during harvest season	Droughts			1				1					1		5
	Prolonged droughts														6
	Flooding														6
	Prolonged flooding														6
	Hailstorms			1	1			2					2		4
	Storms			1	1			2					2		4
	Frosts			1				1					1		5
	Heat waves			1				1					1		5
	Wildfires														6
	Wind gusts				1			1					1		5
	No response		1		1	1	4	1	2	4	1	1	2		
Affected canola production in growth and yield losses	Droughts	2	1	1	3	1		6	2			1	7		1
	Prolonged droughts	2			3	1		5	1			1	5		3
	Flooding	1	1	1	2	1		4	2			1	5		3
	Prolonged flooding	1	1	1	2	1		4	2			1	5		3
	Hailstorms	2	1	1	3	1		6	2			1	7		1
	Storms	2	1	1	3	1		6	2			1	7		1
	Frosts	2	1	1	1	1		5	1			1	5		3
	Heat waves	2	1	1	2	1		6	2			1	6		2
	Wildfires														9
	Wind gusts	2	1	1	1			4	1			1	4		4
	No response						4			4	1	1	2		
Improved canola production in growth and yield gains	Short dry periods - beginning of the growing season		1		3			1	2	1			3		5
	Short dry periods - during growing season			1	2	1		2	1	1			3		5
	Short dry periods – at harvest	1	1	1	2	1		3	2	1		1	4		3
	Greater moisture - beginning of the	2	1	1	3	1		5	2	1		1	6		1

	growing season														
	Greater moisture - during growing season	2	1	1	3	1		5	2	1		1	6		1
	Greater moisture – at harvest	2		1	2			3	1	1		1	4		4
	Late Frosts (Spring)				1					1					8
	Early Frosts (Fall)				1					1					8
	Heat waves - beginning of the growing season	1	1		1			1	1	1			2		6
	Heat waves - during growing season				2					1			1		8
	Heat waves – at harvest	1	1		2			1	2	1		1	2		5
	Wind gusts	1			1			1		1		1			7
	No response						4			4	1	1	2		
Faced and adapted canola productio n	Decreased soil moisture	2		1	3			5	1			1	5		
	Water shortage and availability	2		1	1	1		4	1			1	4		
	Water pricing														
	Carbon loss														
	Input cost/ investment	1	1	1	2			3	2			1	3		
	Soil erosion		1	1	2			2	2				4		
	Pest infection	1	1	1	3			4	2			1	5		
	Diseases	1	1	1	2			3	2			1	4		
	Length of growing season	1			2			2		1		2	2		
	Forest or														

	grass fires													[redacted]	[redacted]
	Change in land use	2						2				1	1	[redacted]	[redacted]
	Poor drainage	1			1			1	1			1	1	[redacted]	[redacted]
	No response						4			4	1	1	2	[redacted]	[redacted]
Consider viable adaptation strategy	Use an irrigation plan													[redacted]	[redacted]
	Use a drainage plan		1	1	2			2	1	1		1	3	[redacted]	[redacted]
	Change planting dates	1	1	1	2			3	2				5	[redacted]	[redacted]
	Plant diversified crops	2	1	1	2	1		5	2			1	6	[redacted]	[redacted]
	Continuous cropping	2			3	1		5	1			1	5	[redacted]	[redacted]
	Reduced tillage	2	1	1	4	1		6	2	1		2	7	[redacted]	[redacted]
	No till seeding	2		1	4			5	1	1		2	5	[redacted]	[redacted]
	Change the drill calibration	1	1	1	1			4				1	3	[redacted]	[redacted]
	Change straight cutting canola to swathing	1	1	1	2			3	1	1		2	3	[redacted]	[redacted]
	Use pod shatter tolerant seed varieties	2	1	1	2			4	1	1		2	4	[redacted]	[redacted]
	Use pod sealant close to maturity													[redacted]	[redacted]
	Use nutrients deep in the soil													[redacted]	[redacted]
	Long-term water manageme	2		1	1			3	1			1	3	[redacted]	[redacted]

	nt														
	Restore or preserve wetlands	1		1	2	1		5				1	4		
	Monitor weather conditions		1		3			2	2				4		
	Use a fertilization plan	2	1	1	4	1		6	2	1		2	7		
	Use a pest and disease control plan	1	1		2			2	1	1		1	3		
	Have an insurance plan			1	3	1		3	1	1		1	4		
	No response						4			4	1				
Implemented /tested the adaptation strategy	Use an irrigation plan				1					1		1			8
	Use a drainage plan	1	1	1	2			3	1	1		2	3		4
	Change planting dates	2	1	1	3	1		6	2			1	7		1
	Plant diversified crops	2		1	3	1		5	1	1		2	5		1
	Continuous cropping	2		1	4	1		6	1	1		2	6		2
	Reduced tillage	2	1	1	4	1		5	2	1		2	6		1
	No till seeding	2	1	1	4	1		6	2	1		2	7		
	Change the drill calibration	2	1	1	3			5	1	1		2	5		
	Change straight cutting canola to 1swathing	2	1	1	3			5	1	1		2	5		
	Use pod shatter tolerant	2		1	3			4	1	1		2	4		

	seed varieties													
	Use pod sealant close to maturity													7
	Use nutrients deep in the soil	1		1	2	1		4		1		2	3	4
	Long-term water manageme nt	1			2			1	1	1		2	1	6
	Restore or preserve wetlands	1			3	1		4		1		2	3	4
	Monitor weather conditions	1		1	4	1		5	1	1		2	5	2
	Use a fertilization plan	2	1	1	4	1		6	2	1		2	7	
	Use a pest and disease control plan	1	1	1	2			3	1	1		2	3	2
	Have an insurance plan	2		1	4	1		6	1	1		2	6	1
	No response						4			4	1	1	2	
Abandoned the adaptation strategy	Use an irrigation plan													8
	Use a drainage plan													4
	Change planting dates													1
	Plant diversified crops													1
	Continuous cropping			1				1					1	2
	Reduced tillage													1

	No till seeding		1				1				1		
	Change the drill calibration												
	Change straight cutting canola to swathing			1		1					1		
	Use pod shatter tolerant seed varieties												
	Use pod sealant close to maturity												7
	Use nutrients deep in the soil												4
	Long-term water manageme nt												6
	Restore or preserve wetlands												4
	Monitor weather conditions												2
	Use a fertilization plan												
	Use a pest and disease control plan												2
	Have an insurance plan	1		1		2				1	1		1
	No response				4			4	1	1	2		
Reason why stopped or	Cost/ investment	2		2		4				1	3		
	Work												

abandoned a strategy	overload													[redacted]	[redacted]
	Failed to perform		1	1	1			2	1				3	[redacted]	[redacted]
	Difficulty													[redacted]	[redacted]
	Worker shortage													[redacted]	[redacted]
	No response				2	1	4			5	1	2	3	[redacted]	[redacted]
Barriers faced when implementing an adaptation strategy	Strategic goals / culture	1		1				2					2	[redacted]	4
	Internal financial resources													[redacted]	2
	Qualified personnel			1	2			2		1		1	2	[redacted]	4
	Information about new products or technology		1		2			1	1	1		1	2	[redacted]	4
	Information about market and profit		1	1	2			2	1	1		1	3	[redacted]	3
	Regulations	1	1		3			2	2	1		2	3	[redacted]	3
	Cost / investment	2	1	1	2			4	1	1		2	4	[redacted]	3
	Access to bank loans				2			1		1		1	1	[redacted]	5
	Access to governmental financial support				2	1		2	1				3	[redacted]	4
	Access to appropriate infrastructure				2	1		1	2				3	[redacted]	6
	No response						4			4	1	1	2	[redacted]	
Drivers faced when implementing an adaptation strategy	Strategic goals / culture		1		2			1	2				3	[redacted]	4
	Internal financial resources		1		1			1	1				2	[redacted]	2
	Qualified personnel	1			1			2					2	[redacted]	4
	Information about new	1			1			2					2	[redacted]	4

	products or technology															
	Information about market and profit	2			1			3				1		2		3
	Regulations			1				1						1		3
	Cost / investment															3
	Access to bank loans			1				1						1		5
	Access to governmental financial support	1		1				2				1		1		4
	Access to appropriate infrastructure															6
	No response						4			4	1	1		2		
See canola production five years from now	Significantly increasing				1			1						1		
	Likely more	1			1			1		1		1		1		
	Similar levels	1	1	1	2	1		4	2			1		5		
	Likely less															
	Stopping completely															
	No response						4			4	1	1		2		

Table S2. Aggregated data gathered through the key informant interviews (n=9).

Nodes	Interviewees (#)	Quotes (#)
1. Adaptation	2	2
1.1 Best Practices	7	17
1.1.1 Climate stress	1	1
1.1.2 Cover Crops	1	1
1.1.3 Crop rotation	6	6
1.1.4 Fertilization	4	13
1.1.5 Intercropping	2	11
1.1.6 Pest and disease control	5	11
1.1.7 Seeding	3	3
1.1.8 Tillage	5	5
1.1.9 Water management	6	23
1.1.10 Weed control	4	10
1.1.11 Yield potential	1	1
1.2 Climate-smart Farms	7	17
1.2.1 Investment	7	11
1.3 Digital or Precision Agriculture	1	5
1.4 Environmental	3	8
1.5 Genetics	9	62
1.6 Research	7	26
2. Climate change	5	5
2.1 Dry	4	11
2.2 Less change	1	2
2.3 More change	5	6
2.4 Rain	6	10
2.5 Snow	0	0
2.6 Temperature	5	6
2.7 Uncertainty	4	6
3. Extreme Event	6	8
3.1 Drought	8	22
3.2 Flood	4	7
3.3Forecast	1	1
3.4 Frost	2	4
3.5 Hail	2	2
3.6 Heat wave	2	4
3.7 Rainstorms	4	5
4. Future	0	0
4.1 Advice	9	22
4.2Canola	8	21
4.3 Concerns	7	20
4.4 Innovation	4	15
4.5 Needs	9	21
5. Impact	0	0
5.1 Canola	1	2
5.1.1 Negative	5	15
5.1.2 Positive	6	11
5.2 Economy	1	2
5.2.1 Negative	7	18
5.2.2 Positive	5	10
5.3 Preparedness	1	1
5.3.1 Market	4	7
5.3.2 Producer	7	20
6. Outreach	1	2
6.1 Communication	7	30
6.1.1 E-mail	1	1
6.1.2 Newsletter	2	2
6.1.3 Phone	1	1
6.1.4 Social Media	5	11

6.1.5 Website	5	6
6.2 Extension Events	4	4
6.2.1 Field Days	3	4
6.2.2 Research presentation	3	6
6.3 Reports	2	4
6.4 Response	7	28
7. Public Policies	7	27
